# **The prevalence of** ***Helicobacter*** ***pylori *is decreasing in Iranian patients **

**Published:** 2015

**Authors:** Sara Ashtari, Mohamad Amin Pourhoseingholi, Mahsa Molaei, Hajar Taslimi, Mohammad Reza Zali

**Affiliations:** 1 *Gastroenterology and Liver Disease Research center, Institute for Gastroenterology and Liver Diseases, Shahid Beheshti University of Medical Science, Tehran, Iran*; 2 *Department of Biostatistics, Shahid Beheshti University of Medical Science, Tehran, Iran*

**Keywords:** Gastritis, Prevalence of* H. pylori*, Intestinal metaplasia, Iranian population

## Abstract

**Aim:**

The objective of this study was to evaluate the time trend of *Helicobacter pylori* (*H. pylori*) prevalence and presence of intestinal Metaplasia over the period of seven years among gastritis Iranian patients.

**Background:**

*H. pylori* is the major causal factor in chronic gastritis. Its acquisition leads to a chronic, usually lifelong, inflammation of the gastric mucosa, which may gradually progress to atrophy with intestinal metaplasia in a significant proportion of infected individuals.

**Patients and methods:**

*H. pylori* and intestinal Metaplasia data among 14,860 consecutive gastritis patients, who referred to the gastrointestinal department of Tehran’s Taleghani Hospital in Iran from 2008 to 2014, was examined by sex and age group. The patients were divided into six age groups (16-30, 30-40, 40-50, 50-60 and >70). The chi-square test was used to compare the qualitative variables.

**Results:**

The overall prevalence rate among patient with *H. pylori* infection was 83.5% (12406/14860) and 11,394 (84.1%) of them were related to the gastritis. The prevalence rate of H*. pylori *among patient with severe gastritis was significantly higher (*P*<0.05) compared to mild and moderate gastritis. In addition, the prevalence of H*. pylori *decreased with age and has been declined in recent years. The presence of intestinal metaplasia increased with age (*P*<0.05).

**Conclusion:**

The results of this study showed that the prevalence of H*. pylori *infection in Iranian population has been declined in recent years; nevertheless it seems to be highly prevalent in Iran. We also find a significant positive relationship between H*. pylori *infection and gastritis. There is no association between sex and infection, however in contrast with the most studies its prevalence decreased with age.

## Introduction

 Gastritis is an inflammation of the stomach and has a high incidence in adults (1). It caused by multiple factors such as *Helicobacter pylori* (*H. pylori*), autoimmune problems, non-steroidal anti-inflammatory medicine, bile reflux, pernicious anemia, alcohol consumption, smoking and other diseases and conditions such as Crohn’s disease and HIV/AIDS. But the main reason for chronic gastritis is *H. pylori* infection (2).

*H. pylori* is a Gram-negative bacteria with strong association with upper gastrointestinal diseases such as peptic ulcer diseases (gastric ulcer, duodenal ulcer) and malignancies (gastric cancer, lymphoma), reported worldwide and in Iran (3, 4). At least half of the world’s population is infected by the *H. pylori*, making it the most widespread infection in the world (5). The acute infection rate of *H. pylori* varies by region. However, *H. pylori* infection is highly prevalent in Asia and developing countries, and multifocal atrophic gastritis (intestinal metaplasia) and gastric adenocarcinomas are more common in these areas (6, 7). The incidence of *H. pylori* is 3-10% of the population every year in developing countries, as compared to 0.5% in developed countries (8). The age at which this bacterium is acquired seems to influence the possible pathologic outcome of the infection: people infected with it at an early age are likely to develop more intense inflammation that may be followed by atrophic gastritis with a higher subsequent risk of gastric ulcer, gastric cancer, or both (9, 10). Acquisition at an older age brings different gastric changes that are more likely to result in duodenal ulcer (11). Infections are usually acquired in early childhood in all countries. However, the infection rate of children in developing nations is higher than in industrialized nations, probably due to poor sanitary conditions, perhaps combined with lower antibiotics usage for unrelated pathologies (12, 13). In developed nations, it is currently uncommon to find infected children, but the percentage of infected people increases with age, with about 50% infected for those over the age of 60 compared with around 10% between 18 and 30 years (14).

*H. pylori* cause chronic active inflammation of the gastric mucosa in the majority of infected patients. In a considerable number of them, this will eventually lead to a loss of gastric glands, and thus the establishment of atrophic gastritis (15). This is associated with the development of intestinal metaplasia and dysplasia (16). These consecutive conditions increase the risk for gastric cancer particularly of the intestinal type (17-19). In accordance with this point of the view, the main objectives of this study are to investigate the prevalence rate of H*. pylori* infection and the presence of intestinal metaplasia among endoscopic patients at Taleghani Hospital, according to age across gender. 

## Patients and Methods

All data for this cross-sectional study were collected from medical records of 14,860 patients suspected of gastritis, who were referred to the department of gastrointestinal of Tehran’s Taleghani Hospital between the years 2008 to 2014 in Iran. All these patients were suspected of gastritis. Therefore, undergoing endoscopic and biopsy they were assessed for gastritis, H*. pylori *and intestinal metaplasia. Finally, we examined the time trend of H*. pylori *prevalence and presence of intestinal metaplasia over the period of seven years in gastritis and non-gastritis patients according to the gender and age groups. The patients were divided into 6 age groups (16-30, 30-40, 40-50, 50-60, 60-70 and >70 years). In addition, these groups were further divided into three minor groups namely young adults (16-40), older adults (40-60) and geriatric group (>60) years. 

**Statistical Analysis**

Data were entered and analyzed using statistical package for social sciences (SPSS) for windows version 21 software (IBM Inc., Chicago, IL, USA). Descriptive statistics and frequency distribution such as mean, standard deviation and percentage were employed. The chi-square test was used to compare the qualitative variables. P< 0.05 was considered as statistically significant. 

## Results

A total of 14,860 patients with a mean age of 48±16.8 (± standard deviation) years old were enrolled in this cross-sectional study and 6953 (46.8%) patients were male. The ranges of the age of patients were 16-93 years. 

A diagnosis of gastritis was established in 13,552 patients, representing approximately (91.2%) of all cases. 

**Table 1:** Gastritis rates according to gender, age and years

		Gastritis		
		No n (%)	Yes n (%)	Total n (100%)
Gender	Male Female	657 (9.4) 651 (8.2)	6296 (90.6) 7258 (91.8)	6953 7907
H*. pylori*	No Yes	296 (12.1) 1012 (8.2)	2158 (87.9) 11394 (91.8)	2454 12406
Intestinal metaplasia	No Yes	1104 (8.9) 204 (8.5)	11364 (91.1) 2185 (91.5)	12471 2389
Age group	16-30 30-40 40-50 50-60 60-70 >70	233 (8.6) 216 (8.7) 209 (7.8) 253 (9.2) 161 (8.7) 139 (9.2)	2492 (91.4) 2253 (91.3) 2482 (92.2) 2495 (90.8) 1697 (91.3) 1365 (90.8)	2725 2469 2691 2748 1858 1504
Years	2008 2009 2010 2011 2012 2013 2014	249 (14.5) 204 (10.7) 279 (8.5) 149 (5.0) 249 (8.9) 108 (9.6) 70 (6.5)	1466 (85.5) 1694 (89.3) 3021 (91.5) 2804 (95.0) 2536 (91.1) 1020 (90.4) 1011 (93.5)	1715 1898 3300 2953 2785 1128 1081

**Table 2:** H*. pylori* rates according to gender, age and years

**H** ***. pylori***				
		**No** **n (%)**	**Yes** **n (%)**	**Total** **N (100%)**
**Gender**	Male Female	1167 (16.8) 1287 (16.3)	5786 (83.2) 6620 (83.7)	6953 7907
**Gastritis**	No Yes	296 (22.6) 2158 (15.9)	1012 (77.4) 11394 (84.1)	1308 13552
**Intestinal** **metaplasia**	No Yes	1840 (14.8) 614 (22.7)	10631 (85.2) 1775 (74.3)	12471 2389
**Age group**	16-30 30-40 40-50 50-60 60-70 >70	435 (16.0) 389 (15.8) 456 (16.9) 506 (18.4) 339 (18.2) 268 (17.8)	2290 (84.0) 2080 (84.2) 2235 (83.1) 2242 (81.6) 1519 (81.8) 1236 (82.2)	2725 2469 2691 2748 1858 1504
**Years**	2008 2009 2010 2011 2012 2013 2014	117 (6.8) 119 (6.3) 108 (3.3) 157 (5.3) 1265 (45.4) 302 (26.8) 386(35.7)	1598 (93.2) 1779 (93.7) 3192 (96.7) 2796 (94.7) 1520 (54.6) 826 (73.2) 695 (64.3)	1715 1898 3300 2953 2785 1128 1081

**Table 3:** Intestinal metaplasia rates according to gender, age and years

**Intestinal metaplasia**				
		**No** **n (%)**	**Yes** **n (%)**	**Total** **N (100%)**
Gender	Male Female	5779 (83.1) 6692 (84.5)	1174 (16.9) 1215 (15.4)	6953 7907
Gastritis	No Yes	1104 (84.4) 11367 (83.9)	204 (15.6) 2185 (16.1)	1308 13552
H*. pylori*	No Yes	1840 (75.0) 10631 (85.7)	614 (25.0) 1775 (14.3)	2454 12406
Age group	16-30 30-40 40-50 50-60 60-70 >70	2462 (90.3) 2195 (88.9) 2304 (85.6) 2276 (82.8) 1441 (77.6) 1066 (70.9)	263 (9.7) 274 (11.1) 397 (14.4) 472 (17.2) 4171 (22.4) 438 (29.1)	2725 2469 2691 2748 1858 1504
Years	2008 2009 2010 2011 2012 2013 2014	1380 (80.5) 1552 (81.8) 2894 (87.7) 2391 (81.0) 2355 (84.6) 963 (85.4) 936 (86.6)	335 (19.5) 346 (19.5) 406 (12.3) 582 (19.0) 430 (15.4) 165 (14.6) 145 (13.4)	1715 1898 3300 2953 2785 1128 1081

Gastritis patients detected as mild with 4,479 (30.1%), moderate with 7,430 (50.0%) and severe form with 1,643 (11.1%). There was no association between gastritis and sex. Gastritis was present alike in almost age groups and in during the 7 years. Table 1 shows the situation of gastritis according to the gender, various ages and years.

The overall prevalence of *H. pylori* infection in this study was 83.5% (12406/14860), we also found the strong association (*P*<0.05) between H*. pylori* infection and gastritis with 84.1% (11,394/13,552) prevalence of H*. pylori* infection in gastritis patients. The prevalence rate of H*. pylori *among patient with severe gastritis was significantly higher (*P*<0.05), compared to mild and moderate gastritis. The prevalence of H*. pylori* infection decreased with age and it has also declined in recent years (*P*<0.05). Table 2 shows the prevalence rate of H*. pylori *according to the gender, various age and years.

Totally, the presence of intestinal metaplasia in these patients was 2389, representing approximately (16.1%). The rate of intestinal metaplasia was significantly higher (*P*<0.05) in H*. pylori* infected patients. Besides it was significantly (*P*<0.05) more common in severe gastritis patients. The age range further categorized into 3 groups including: young adults (16-40), older adults (40-60) and geriatric (>60 years). The rate of intestinal metaplasia increased with age. The rate among geriatric groups (>60 year) was significantly (*P*<0.05) higher than younger and older adults. Also, intestinal metaplasia has been decreased through these years. Table 3 shows the presence of intestinal metaplasia according to the gender, various ages and years. 

## Discussion

In this study, the prevalence of H*. pylori *infection and presence of intestinal metaplasia was determined among the Iranian population, indicating that the prevalence of H*. pylori *infection is still high among Iranian patients. However, its rate is slightly declining through recent years. 

Several studies have reported different prevalence rate of H*. pylori *infection between countries (20, 21), which might be due to diverse contributing factors including socioeconomic status, geographical or living conditions, as well as ethnicity or location of each population (3, 22). 

Similar to several studies from Asia and Middle East including China, South Korea, Iran, and Kuwait, our research showed that the prevalence of *H. pylori* has declined in recent years (21, 23-26), which might be related to the human host factors as well as socioeconomic and hygiene factors (27). It appears that acquisition and transmission of *H. pylori* can be prevented to a large extent by following improved hygienic practices and standard of living (28). In all participants in this study, the prevalence of H*. pylori* was 83.5%, and also the prevalence rate of H*. pylori *infection only in gastritis patients was 84.1%, which was similar to some other studies that reported the H*. pylori *infection prevalence in Shiraz, Ardebil and north of Iran more than 85% (4,6, 29). We analyzed the presence of H*. pylori *infection among patients with different ages and found that the prevalence of H*. pylori *infection decreased with age as similar as other studies (30) However, several studies showed that the prevalence of H*. pylori *infection increased with age in developing and developed countries (31-33). 

Intestinal metaplasia represents a phenotypic change from the normal epithelial cells of the gastric mucosa to an intestinal phenotype (30, 34). It is considered to be an advanced stage of atrophy because the original glands are replaced by metaplastic glands and chronologically, the metaplastic glands appear after the gastric glands are lost (35-37). In the present study, the percent of intestinal metaplasia was 16.1% and also the presence of intestinal metaplasia in patients with H*. pylori *infection was 14.3%. Based on our results, the presence of intestinal metaplasia has been declined in recent years, but in contrast with H*. pylori *infection, the prevalence of intestinal metaplasia increased with age. In this study we just presented the data of patients upper 16 years old because patients lower than 16 years did not undergo endoscopic in Taleghani Hospital. Therefore, further research should be done to find the rate and prevalence of H*. pylori *infection in Iranian children and provide a full spectrum of this infection for Iranian population.

## References

[1] Rostami-Nejad M, Villanacci V, Mashayakhi R, Molaei M, Bassotti G, Zojaji H (2009). Celiac disease and Hp infection association in Iran. Rev Esp Enferm Dig.

[2] National Institute of Diabetes and Digestive and Kidney Diseases Gastritis.

[3] Niknam R, Seddigh M, Fattahi MR, Dehghanian A, Mahmoudi L (2014). Prevalence of Helicobacter pylori in patients with dyspepsia. Jundishapur J Microbiol.

[4] Massarrat S, Saberi-Firoozi M, Soleimani A, Himmelmann GW, Hitzges M, Keshavarz H (1995). Peptic ulcer disease, irritable bowel syndrome and constipation in two populations in Iran. Eur J Gastroenterol Hepatol.

[5] Pounder RE, Ng D (1995). The prevalence of Helicobacter pylori infection in different countries. Aliment Pharmacol Ther.

[6] Malekzadeh R, Sotoudeh M, Derakhshan MH, Mikaeli J, Yazdanbod A, Merat S (2004). Prevalence of gastric precancerous lesions in Ardabil, a high incidence province for gastric adenocarcinoma in the northwest of Iran. J Clin Pathol.

[7] Nabwera HM, Nguyen-Van-Tam JS, Logan RF, Logan RP (2000). Prevalence of Helicobacter pylori infection in Kenyan schoolchildren aged 3-15 years and risk factors for infection. Eur J Gastroenterol Hepatol.

[8] Go MF (2002). Review article: natural history and epidemiology of Helicobacter pylori infection. Aliment Pharmacol Ther.

[9] Genta RM, Sonnenberg A (2015). Helicobacter-negative gastritis: a distinct entity unrelated to Helicobacter pylori infection. Aliment Pharmacol Ther.

[10] Kara N, Urganci N, Kalyoncu D, Yilmaz B (2014). The association between Helicobacter pylori gastritis and lymphoid aggregates, lymphoid follicles and intestinal metaplasia in gastric mucosa of children. J Paediatr Child Health.

[11] Ghasemi-Kebria F, Ghaemi E, Azadfar S, Roshandel G (2013). Epidemiology of Helicobacter pylori infection among Iranian children. Arab J Gastroenterol.

[12] Poddar U, Yachha SK (2007). Helicobacter pylori in children: an Indian perspective. Indian Pediatr.

[13] Khedmat H, Karbasi-Afshar R, Agah S, Taheri S (2013). Helicobacter pylori infection in the general population: a Middle Eastern perspective. Caspian J Intern Med.

[14] Rowland M, Daly L, Vaughan M, Higgins A, Bourke B, Drumm B (2006). Age-specific incidence of Helicobacter pylori. Gastroenterology.

[15] Benberin V, Bektayeva R, Karabayeva R, Lebedev A, Akemeyeva K, Paloheimo L (2013). Prevalence of H. pylori infection and atrophic gastritis among symptomatic and dyspeptic adults in Kazakhstan. A hospital-based screening study using a panel of serum biomarkers. Anticancer Res.

[16] Sozzi M, Valentini M, Figura N, De Paoli P, Tedeschi RM, Gloghini A (1998). Atrophic gastritis and intestinal metaplasia in Helicobacter pylori infection: the role of CagA status. Am J Gastroenterol.

[17] Sipponen P (1994). Gastric cancer--a long-term consequence of Helicobacter pylori infection?. Scand J Gastroenterol Suppl.

[18] Sipponen P, Hyvarinen H (1993). Role of Helicobacter pylori in the pathogenesis of gastritis, peptic ulcer and gastric cancer. Scand J Gastroenterol Suppl.

[19] Uemura N, Okamoto S, Yamamoto S, Matsumura N, Yamaguchi S, Yamakido M (2001). Helicobacter pylori infection and the development of gastric cancer. N Engl J Med.

[20] Chen J, Bu XL, Wang QY, Hu PJ, Chen MH (2007). Decreasing seroprevalence of Helicobacter pylori infection during 1993-2003 in Guangzhou, southern China. Helicobacter.

[21] Farshad S, Japoni A, Alborzi A, Zarenezhad M (2010). Changing prevalence of Helicobacter pylori in south of Iran. Iran J Clin Infect Dis.

[22] Bastos J, Peleteiro B, Barros R, Alves L, Severo M, de Fatima Pina M (2013). Sociodemographic determinants of prevalence and incidence of Helicobacter pylori infection in Portuguese adults. Helicobacter.

[23] Alazmi WM, Siddique I, Alateeqi N, Al-Nakib B (2010). Prevalence of Helicobacter pylori infection among new outpatients with dyspepsia in Kuwait. BMC Gastroenterol.

[24] Yim JY, Kim N, Choi SH, Kim YS, Cho KR, Kim SS (2007). Seroprevalence of Helicobacter pylori in South Korea. Helicobacter.

[25] Goh KL (1997). Prevalence of and risk factors for Helicobacter pylori infection in a multi-racial dyspeptic Malaysian population undergoing endoscopy. J Gastroenterol Hepatol.

[26] Amini E, Keshteli AH, Jazi MS, Jahangiri P, Adibi P (2012). Dyspepsia in Iran: SEPAHAN Systematic Review No. 3. nt J Prev Med.

[27] Lim SH, Kwon JW, Kim N, Kim GH, Kang JM, Park MJ (2013). Prevalence and risk factors of Helicobacter pylori infection in Korea: nationwide multicenter study over 13 years. BMC Gastroenterol.

[28] Ahmed KS, Khan AA, Ahmed I, Tiwari SK, Habeeb A, Ahi JD (2007). Impact of household hygiene and water source on the prevalence and transmission of Helicobacter pylori: a South Indian perspective. Singapore Med J.

[29] Shafii M, Nikzad SE, Kasiri H, Naghipour M (2008). Histopathological evaluation of chronic gastritis with and without Helicobacter pylori colonization: a study from Iran. Malays J Pathol.

[30] Chen S, Ying L, Kong M, Zhang Y, Li Y (2013). The prevalence of Helicobacter pylori infection decreases with older age in atrophic gastritis. Gastroenterol Res Pract.

[31] Logan RP, Walker MM (2001). ABC of the upper gastrointestinal tract: Epidemiology and diagnosis of Helicobacter pylori infection. BMJ.

[32] Veldhuyzen van Zanten SJ, Pollak PT, Best LM, Bezanson GS, Marrie T (1994). Increasing prevalence of Helicobacter pylori infection with age: continuous risk of infection in adults rather than cohort effect. J Infect Dis.

[33] Bruden DL, Bruce MG, Miernyk KM, Morris J, Hurlburt D, Hennessy TW (2011). Diagnostic accuracy of tests for Helicobacter pylori in an Alaska Native population. World J Gastroenterol.

[34] Ebule IA, Longdoh AN, Paloheimo IL (2013). Helicobacter pylori infection and atrophic gastritis. Afr Health Sci.

[35] Dirnu R, Secureanu FA, Neamtu C, Totolici BD, Pop OT, Mitrut P (2012). Chronic gastritis with intestinal metaplasia: clinico-statistical, histological and immunohistochemical study. Rom J Morphol Embryol.

[36] Dobrilla G, Benvenuti S, Amplatz S, Zancanella L (1994). Chronic gastritis, intestinal metaplasia, dysplasia and Helicobacter pylori in gastric cancer: putting the pieces together. Ital J Gastroenterol.

[37] Mansour-Ghanaei F, Joukar F, Soati F, Mansour-Ghanaei A, Atrkar-Roushan Z (2013). Outcome of intestinal metaplasia in gastric biopsy of patients with dyspepsia in Guilan Province, North Iran. Asian Pac J Cancer Prev.

